# Motion-Aware Correlation Filters for Online Visual Tracking

**DOI:** 10.3390/s18113937

**Published:** 2018-11-14

**Authors:** Yihong Zhang, Yijin Yang, Wuneng Zhou, Lifeng Shi, Demin Li

**Affiliations:** College of Information Science and Technology, Engineering Research Center of Digitized Textile & Fashion Technology, Ministry of Education, DongHua University, Shanghai 201620, China; wnzhou@dhu.edu.cn (W.Z.); 2161238@mail.dhu.edu.cn (L.S.); deminli@dhu.edu.cn (D.L.)

**Keywords:** visual tracking, correlation filters, motion-aware, adaptive update strategy, confidence response map

## Abstract

The discriminative correlation filters-based methods struggle deal with the problem of fast motion and heavy occlusion, the problem can severely degrade the performance of trackers, ultimately leading to tracking failures. In this paper, a novel Motion-Aware Correlation Filters (MACF) framework is proposed for online visual object tracking, where a motion-aware strategy based on joint instantaneous motion estimation Kalman filters is integrated into the Discriminative Correlation Filters (DCFs). The proposed motion-aware strategy is used to predict the possible region and scale of the target in the current frame by utilizing the previous estimated 3D motion information. Obviously, this strategy can prevent model drift caused by fast motion. On the base of the predicted region and scale, the MACF detects the position and scale of the target by using the DCFs-based method in the current frame. Furthermore, an adaptive model updating strategy is proposed to address the problem of corrupted models caused by occlusions, where the learning rate is determined by the confidence of the response map. The extensive experiments on popular Object Tracking Benchmark OTB-100, OTB-50 and unmanned aerial vehicles (UAV) video have demonstrated that the proposed MACF tracker performs better than most of the state-of-the-art trackers and achieves a high real-time performance. In addition, the proposed approach can be integrated easily and flexibly into other visual tracking algorithms.

## 1. Introduction

Visual object tracking is one of the most popular fields in computer vision for its wide applications including unmanned vehicles, video surveillance, UAV, and human-computer interaction, where the goal is to estimate the locus of the object given only by an initial bounding box from the first frame in the video stream [1]. Although significant progress has been achieved in recent decades, accurate and robust online visual object tracking is still a challenging problem due to the parameters of fast motion, scale variations, partial occlusions, illumination changes and background clutters [2].

In recent decades, visual object tracking has been widely studied by researchers resulting in a large body of work. The most relevant works, which had been tested on the benchmark datasets of OTB-50 [3], OTB-100 [4], and the Visual Object Tracking benchmarks of VOT-2014 [5], and VOT-2016 [6], are discussed below.

In general, visual object tracking approaches can be broadly classified into two categories, generative methods [7,8,9,10,11,12,13] and discriminative methods [14,15,16,17,18,19,20,21,22,23,24,25]. The generative methods use the features extracted from the previous frame to establish the appearance model of the target, and then search for the most similar region and locate the position of the target in the current frame. Robust Scale-Adaptive Mean-Shift for Tracking (ASMS) [8] and Distractor-Aware Tracker (DAT) [7] are the two most representative trackers in generative methods. ASMS is a real-time algorithm using the color histogram features for visual tracking where a scale estimation strategy is added to the classical mean-shift framework. However, it is easily distracted by similar objects in the surroundings. The improved method DAT is a distractor-aware tracking algorithm based on the color probabilistic model of the foreground and the background. It uses the Bayesian method to determine the probability of each pixel belonging to the foreground or background to suppress similar objects in the vicinity. However, these methods make the trend of scale shrink for the use of color features where the edge pixels are always overlooked. Meanwhile, the discriminative approaches which are also called as ‘track-by-detection methods’ are popular for their high accuracy, robustness, and real-time performance. These methods employ machine-learning techniques to train classifiers by numbers of positive and negative samples extracted from the previous frame, and then use the trained classifiers to find the optimal area of the target and locate the position of the target. Among the discriminative approaches, the Discriminative Correlation Filter-based (DCF-based) approach is one of the most popular approach.

### 1.1. DCF-Based Trackers

Lately, Discriminative Correlation Filters (DCFs) have been extensively applied to visual object tracking in computer vision. It was introduced into the visual tracking fields by Bolme and colleagues in the article visual object tracking using adaptive correlation filters [1]. It named by Minimum Output Sum of Squared Error (MOSSE) which produced astonishing results with tracking speed reaching about 700 Frames Per Second (FPS). Thereafter, numerous improved algorithms [14,15,16,17,26,27,28] based on DCFs have been published with accurate and robust tracking results by sacrificing the tracking speed. The DCF technique is a computationally efficient process in the frequency domain transformed by fast Fourier transform (FFT) [1,29,30]. It is a supervised method for learning a linear classifier or a linear regressor, which trains and updates DCFs online with only one real sample given by the bounding box and various synthetic samples generated by cyclic shift windows. Then the trained DCFs are used to detect the position and scale of the target in the subsequent frame. 

Currently, DCF-based methods such as Discriminative Scale Space Tracking (DSST) [16], Fast Discriminative Scale Space Tracking (FDSST) [16], and Spatially Regularized Discriminative Correlation Filters (SRDCF) [26] have demonstrated excellent performance on the popular benchmarks OTB-100 [4], VOT-2014 [5], and VOT-2016 [6]. The DSST trains separate translation and scale correlation filters by the Histogram of Oriented Gradient (HOG) features. And the trained correlation filters are used to respectively detect the position and scale of the target. Then the improved FDSST use the principal component analysis (PCA) method to reduce the dimension of the features to speed up the DSST. However, all these methods detect the target by exploiting a limited search region usually smaller than the whole figure. Although it can reduce computational costs, it can result in tracking failures when the target moves out of the search region due to fast motion or heavy occlusion.

Generally, to reduce the computation costs, the standard DCF-based method tracks the target using a padding region which is several times larger than the target but with size limited. In addition, it multiplies a cosine window with the same size of padding region to emphasize on the target [1,14,16,17,26,31]. Despite its excellent properties, the DCF approach cannot detect the position of the target correctly when the target moves to the boundaries of the padding region. Additionally, it fails to track the target when the target moves out of the padding region due to fast motion or heavy occlusion. The dilemma between a larger padding region which is more computationally expensive and a smaller padding region which lacks the ability to track the target, significantly influences the capabilities of the DCF methods. Furthermore, most of the state-of-the-art DCF-based methods [7,16,17,26,27,32] estimate the scale of the target by using a limited number of scales of various sizes. It results in scale tracking failures when the scale changes significantly due to the fast motion. The dilemma between the exhaustive scale search strategies resulting in higher computational costs and the finite number of scale estimation method leading to failures of scale estimation, severely reduced the robustness of the DCF algorithm. Resolving these two dilemmas are the main aims of the present paper.

### 1.2. Solutions to the Problem of Fast Motion

To solve the dilemmas, a concise and efficient instantaneous motion estimation method (which is implemented by the differential velocity and acceleration between frames) is applied to predict the possible position and scale of the detected target. Nevertheless, the noises existing in the detected results can dramatically affect the performance of this method. For eliminating the noises of the detected results, we prefer to choose the optimal Kalman filter [33,34,35,36,37] which is a highly efficient autoregressive filter. It can estimate the state of a dynamic system in a combination of many uncertainties. In addition, it is a powerful and versatile tool which is appropriate for changing constantly systems. In recent decades, Kalman filters have been widely used in the field of visual object tracking due to the advantage of a small memory footprint (just retaining the previous state) and computational efficiency. It is ideal for real-time problems and embedded systems [33,34,36,38,39,40,41], which can improve the performance of trackers without sacrificing the real-time property.

### 1.3. Our Contributions

This paper, inspired by the works [16,42,43,44,45], proposes a novel Motion-Aware Correlation Filters (MACF) visual tracker which aims to solve the two dilemmas described in Section 1.1. The proposed approach initializes the joint instantaneous motion estimation Kalman filters by using the parameters of the bounding box given by the first frame. Then the improved Kalman filters are used to predict the probable position and scale of the target in the subsequent frame. This makes the target near the center of the padding region which improves the robustness and accuracy of the tracker. The DCFs-based tracker [16] is chosen as the fundamental framework to train correlation filters to detect the location and scale of the target based on the predicted results. For the convenience of computation and integration, the Kalman Filters are decomposed into two parts including a two-dimensional in-plane motion estimation filter and a one-dimensional depth motion estimation filter [46]. In addition, a novel function is proposed to compute the confidence of the response map to determine whether to update the correlation filters. The lower the confidence score is, the higher probability the model is corrupted. Hence, the score below the set threshold means that the target has been occluded or has changed greatly. Then, the learning rate is reduced according to the confidence of the response map to overcome the problem. In this paper, all the implementation and testing codes are all open source in the following Github web: https://github.com/YijYang/MACF.git.

In summary, the main contributions of this paper include:

1. A novel tracking framework named MACF which corrects the padding region using motion cues predicted by separated joint instantaneous motion estimation Kalman filters, one for in-plane position prediction and the other for scale prediction;
2. An attractive confidence function of the response map to identify the situation where the target is occluded or corrupted and an adaptive learning rate to prevent the model from being corrupted.
3. Qualitative and quantitative experiments on OTB-50, OTB-100 and UAV video have demonstrated that our approach outperforms most of the state-of-the-art trackers.

## 2. The Reference Tracker

**Figure 1:**
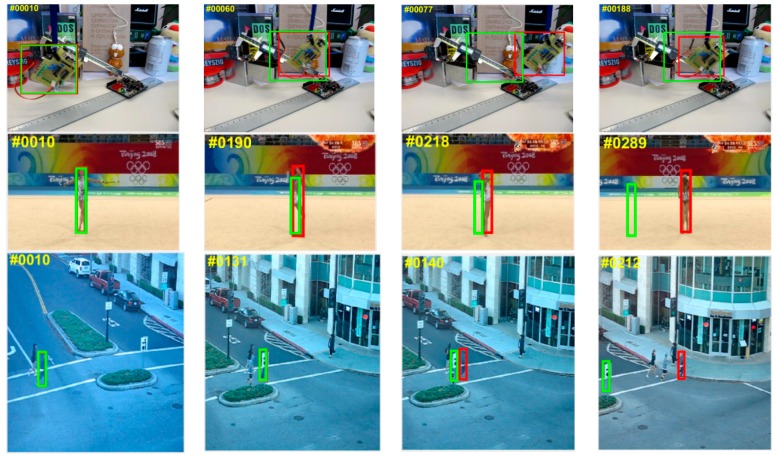
The comparison of tracking results between our MACF tracker (in red) and the standard FDSST tracker (in green) in three sequences on OTB-100 benchmark. Our tracker performs better than FDSST in the example frames which are shown from the “Board” of fast motion (**top** row), “Gym1” of scale change (**middle** row) and “Human4.2” of heavy occlusion (**bottom** row) videos.

In this section, the reference framework of the FDSST tracker is introduced in detail. In contrast to the FDSST, the proposed MACF tracker has been improved on this baseline tracker and achieved a significant progress on the benchmarks as shown in Figure 1.

The FDSST tracker is chosen as the baseline of the proposed MACF framework due to its superior performance on VOT-2014. Unlike the other DCFs-based methods, the FDSST tracker learns 1-dimensional scale estimation correlation filters and 2-dimensional translation estimation correlation filters separately, which is implemented by adjusting the feature extraction procedure only for each case [16]. The objective function of correlation filter f can be denoted as follows including a response score function (1) and an $L^{2}$ error function (2) with *t* samples:

(1) $$S_{f}\left(x\right)=\sum_{l=1}^{d}x^{l}*f^{l}$$

(2) $$\varepsilon \left(f\right)=\sum_{k=1}^{t}{\left\|S_{f}(x_{k})-g_{k}\right\|}^{2}+\lambda \sum_{i=1}^{d}{\left\|f^{l}\right\|}^{2}$$

where * denotes circular convolution operation and x denotes the HOG features extracted from the target samples. In function (1), *l* indicates the *l*-dimensional HOG features and *d* represents the total dimension of the HOG features. In function (2), the desired output $g_{k}$ presents a 2-dimentional Gaussian function with the same size of f and x, and *k* denotes the *k* represents the *k*th sample of the input. The second term in Equation (2) is a regularization term with a parameter λ (λ≥0). 

The function (2) is a linear least square problem which can be solved efficiently in frequency domain transformed by FFT. Therefore, through minimizing the function (2), the final solution can be computed by Equation (5), which is equivalent to solving a system of linear equations as follows:

(3) $$A_{t}^{l}=\bar{G}F^{l}$$

(4) $$B_{t}=\sum_{k=1}^{d}{\bar{X}}_{t}^{k}X_{t}^{k}+\lambda$$

(5) $$F_{t}^{l}=\frac{A_{t}^{l}}{B_{t}},l=1,2,\ldots ,d$$

where the capital letters denote the FFT and $F_{t}$ denotes the correlation filter in the Fourier domain. In Equations (3) and (4), $A_{t}$ denotes the numerator of the filter, and $B_{t}$ denotes the denominator of the filter. The overbar of $\bar{X}$ denotes the complex conjugation of X.

For computational efficiency, the size of the filter $F_{t}$ is the same as the padding region which is twice the size of the bounding box. An optimal update strategy is utilized to the numerator $A_{t}$ in Equation (6) and the denominator $B_{t}$ in Equation (7) of the filter $F_{t}$ with a new sample feature $X_{t}$ as follows:

(6) $$A_{t}^{l}=(1-\eta_{0})A_{t-1}^{l}+\eta_{0}\bar{G}F^{l}$$

(7) $$B_{t}=(1-\eta_{0})B_{t-1}+\eta_{0}\sum_{k=1}^{d}{\bar{X}}_{t}^{k}X_{t}^{k}$$

where the scalar $\eta_{0}$ is a parameter of the learning rate.

To detect the variations of position $P_{t}$ and scale $S_{t}$ of the target, the FDSST firstly learns a 2-dimensional DCF for position estimation and then learns a 1-dimensional DCF for scale estimation. The responding scores $y_{t}$ for a new frame can be formulated by function (8).

(8) $$y_{t}=F^{-1}\left\{\frac{\sum_{l=1}^{d}{\bar{A}}_{t-1}^{l}Z^{l}}{B_{t-1}+\lambda }\right\}$$

where $Z^{l}$ denotes the l-dimensional HOG features extracted from the frame of pending detection. $F^{-1}$ represents the Inverse Fast Fourier Transform (IFFT). In Algorithm 1, the capital letter $Y_{t,trans}$ denotes the response scores of translation model and $Y_{t,scale}$ denotes the response scores of scale model. By computing the IFFT, the obtained spatial distribution of the response map is used to determine the spatial location and scale of the target.

Consequently, the position or the scale of the target is determined by the maximal value of the scores y of the corresponding DCFs. In addition, to ultimately reduce the computational costs, the principal component analysis (PCA) method is utilized to decrease the dimension of Histogram of Oriented Gradient (HOG) features. For further details see references [5,6].

## 3. Our Approach

In this section, two different approaches for motion estimation of the target is introduced, including the instantaneous motion estimation method and Kalman Filters-based motion estimation method. Then the proposed MACF framework is introduced in detail. Firstly, the Joint instantaneous motion estimation Kalman filters for motion prediction are investigated. Secondly, an update scheme with an adaptive learning rate to prevent the model corrupted by heavy occlusion or fast motion is presented. Finally, the algorithm framework of MACF is described in Algorithm 1.

### 3.1. Instantaneous Motion Estimation between Three Adjacent Frames

**Figure 2:**
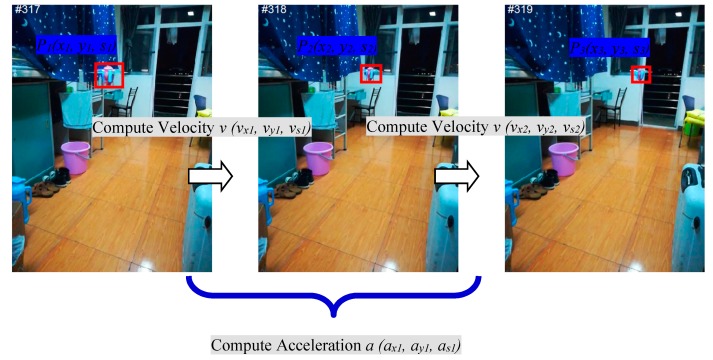
Illustrating of instantaneous motion estimation on the test sequence of UAV.

A single scheme for incorporating motion estimation is to estimate instantaneous velocity and acceleration between three contiguous frames as shown in Figure 2. Firstly, this method initializes the parameters of position and scale to ($x_{1}$, $y_{1}$, $s_{1}$), and sets the velocity and acceleration of the *x*-axis, *y*-axis, and *z*-axis ($v_{x_{1}}$, $v_{y_{1}}$, $v_{s_{1}}$), ($a_{x_{1}}$, $a_{y_{1}}$, $a_{s_{1}}$) to (0, 0, 0) in the first frame. Secondly, these parameters are utilized to predict the possible region of the target by Equation (11) in the second frame. Then the FDSST is used to detect the position ($x_{2}$, $y_{2}$) and the scale $s_{2}$ of the target to update ($v_{x_{2}}$, $v_{y_{2}}$, $v_{s_{2}}$) by function (9). In the third frame, the accelerations ($a_{x_{2}}$, $a_{y_{2}}$, $a_{s_{2}}$) are updated by function (10). Finally, it continuously predicts and detects the location and scale of the target until the last frame of the video stream.

(9) $$\left\{ \begin{array}{l} v_{x_{t}}=x_{t}-x_{t-1}\\ v_{y_{t}}=y_{t}-y_{t-1}\\ v_{s_{t}}=s_{t}-s_{t-1} \end{array}\right.$$

(10) $$\left\{ \begin{array}{l} a_{x_{t}}=v_{x_{t}}-v_{x_{t-1}}\\ a_{y_{t}}=v_{y_{t}}-v_{y_{t-1}}\\ a_{s_{t}}=v_{s_{t}}-v_{s_{t-1}} \end{array}\right.$$

(11) $$\left\{ \begin{array}{l} Px_{t+1}=x_{t}+v_{x_{t}}\cdot \Delta t+0.5\cdot a_{x_{t}}\cdot \Delta t^{2}\\ Py_{t+1}=y_{t}+v_{y_{t}}\cdot \Delta t+0.5\cdot a_{y_{t}}\cdot \Delta t^{2}\\ Ps_{t+1}=s_{t}+v_{s_{t}}\cdot \Delta t+0.5\cdot a_{s_{t}}\cdot \Delta t^{2} \end{array}\right.$$

where Δt denotes time step, Δt=1 is used to facilitate the calculation, (x, y, s) denote the results of detection, and (Px, Py, Ps) denote the results of the prediction.

However, this approach can be affected easily by the noise of the detected results. In addition, the basic tracker FDSST has quite a fine scale detection. Hence, the error scale estimation, which is caused by measurement noise, probably leads to tracking failures.

### 3.2. Kalman Filters-Based Motion Estimation

For high accuracy of the motion prediction, Kalman Filters serve as a strategy of motion estimation [38,39]. Assuming that the motion model of the target is a constant acceleration model, the motion model can be described by the linear stochastic differential functions as follows:

(12) P(t)=AP(t−1)+BM(t)+W(t)

(13) Z(t)=HP(t)+V(t)

In the above two equations, P(t) is the target state of the *t*-th frame of the video sequence, and M(t) is the motion model of the target in the *t*-th frame. In function (12), A and B are the parameters of the motion model. In Formula (13), Z(t) is the measured value of the target state of the *t*-th frame and H is the parameter of the measurement system. In the two equations, W(t) and V(t) represent the process and measured noise respectively and they are assumed to be White Gaussian Noise. Their covariances are Q and R which are assumed not to change with the system state. Q and R respectively represent the confidence of the predicted value and the measured value. It can affect the weight of the predicted value and the measured value through affecting the value of the Kalman gain in the Equation (16). When the value of R is larger, the confidence of the measured value is smaller.

#### 3.2.1. Prediction

For a system which satisfies the above conditions, the Kalman Filter is the optimal information processor. Firstly, the motion model of the target is used to separately predict the position and scale of the target in the next state. Secondly, the current system state is t, the function (14) can be used to predict the position or scale in the current state based on the previous state P(t−1|t−1) of the target. Finally, the current covariance of C(t−1|t−1) can be updated by Equation (15).

(14) P(t|t−1)=AP(t−1|t−1)+BM(t) 

(15) $$C\left(t|t-1\right)=AC\left(t-1|t-1\right)A^{\prime }+Q\;$$

where, P(t|t−1) is the current predicted position or scale of the target, and P(t−1|t−1) is the result of the previous state optimization. In Equation (15) C(t|t−1) is the covariance corresponding to P(t|t−1) and C(t−1|t−1) is covariance corresponding to P(t−1|t−1). In formula (15), $A^{\prime }$ denotes the transpose matrix of A and Q is the covariance of the motion model which has been set in the first frame.

#### 3.2.2. Measurement and Correction

The position and scale of the target detected by FDSST mentioned in Section 3.1 is used as the measurement value Z(t). Combined with the prediction result P(t|t−1), the measurement value Z(t), and the Kalman gain calculated by Equation (16), the optimal estimate of the current position P(t|t) is achieved using Equation (17).

(16) $$Kg\left(t\right)=\frac{C\left(t|t-1\right)H^{\prime }}{HC\left(t|t-1\right)H^{\prime }+R}\;$$

(17) P(t|t)=P(t|t−1)+Kg(t)[Z(t)−HP(t|t−1)] 

where Kg(t) is the Kalman gain in current frame and $H^{\prime }$ denotes the transpose matrix of H, and R denotes the measuring error. In short, Q and R respectively represent the confidence of the predicted value and the measured value and can affect the weight of the predicted value and the measured value by affecting the value of the Kalman gain Kg(t). The larger the R, the less the confidence is the measured value.

To keep the Kalman filter running until the last frame of the video streaming [47], the new covariance of C(t|t) is updated by function (18).

(18) C(t|t)=[I−Kg(t)H]C(t|t−1) 

where, I is a unit matrix.

### 3.3. Motion-Aware in Our Framework

Assuming that White Gaussian Noises exist in the measured velocity and acceleration in Equations (9) and (10), the measured results are utilized to predict the position of the target by a linear Equation (11). Obviously, the predictions include the White Gaussian Noises which potentially result in tracking failures. Therefore, the joint instantaneous motion estimation Kalman Filters are utilized to filter out the noise of the predicting results. It means that the predicted values by Equation (11) are taken as the observed input value of the Kalman filter and then output an optimal prediction by Equation (17).

**Figure 3:**
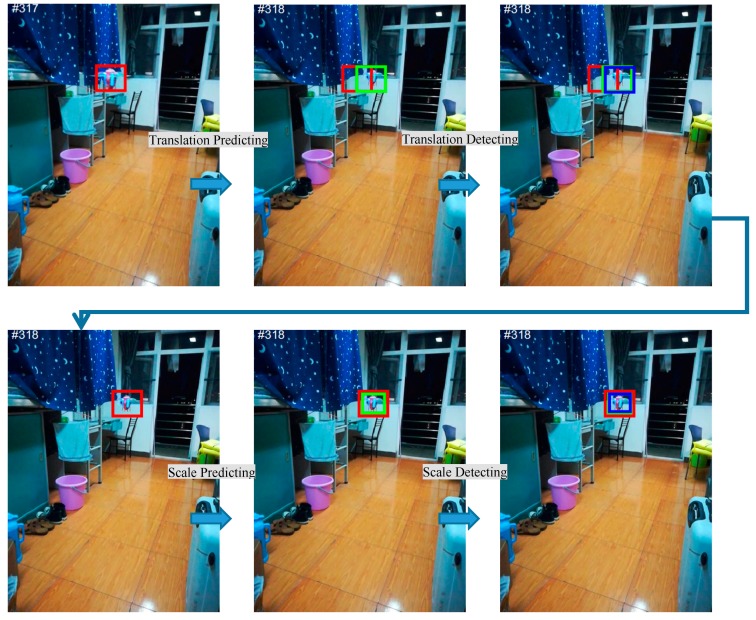
Visualization of the separate translation and scale prediction and detection on the video sequence of UAV. The previous position and scale are indicated by red bounding box, and the predicted position and scale are denoted in green, and the detected position and scale are shown with blue bounding box.

As mentioned in Section 3.2, the instantaneous motion estimation method is affected greatly by the noise, but it can deal with the nonlinear motion model. However, the Kalman Filter filters out the noises, but cannot solve the nonlinear motion model. Hence, for achieving the advantages of both methods, the two methods are combined for Motion estimation of the target. Additionally, for convenient and efficient computation, the optimal Kalman Filters are set up separately for position and scale prediction as shown in Figure 3.

(I) The position prediction filter is responsible for the prediction of the target location and noise filtering. First, motion parameters ($v_{x_{t-1}}$, $v_{y_{t-1}}$, $a_{x_{t-1}}$, $a_{y_{t-1}}$) are employed in the previous frame to predict the translation $PP_{t}$ ($Px_{t}$, $Py_{t}$) of the target in the next frame through Equation (11). After that, the two-dimensional Kalman position filter is utilized to eliminate the noises of the prediction by function (17).

(II) The scale prediction filter is employed to predict accurately and reliably the scale of the target by filtering noises. The prediction parameters ($v_{x_{s-1}}$, $a_{s_{t-1}}$) are first utilized in the front frame to predict the scale $Ps_{t}$ of the target in the following frame by Equation (11). Afterwards, the one-dimensional Kalman scale filter is employed to remove the noises of the prediction by function (17).

### 3.4. Position and Scale Detection

The two-dimensional translation correlation filter $F_{t,trans}$ of the FDSST (described in Section 3.1) is used to detect the position of the target in a small padding region based on the filtered predictions. Then, the results of detection ($x_{t}$, $y_{t}$) is utilized to update the in-plane motion model parameters ($v_{x_{t}}$, $v_{y_{t}}$, $a_{x_{t}}$, $a_{y_{t}}$) via Equations (9) and (10). Similarly, for estimating the scale of the target, the scale correlation filter $F_{t,scale}$ is utilized to correct the scale of the target on the foundation of the predicted scale. Then, the estimated scale $s_{t}$ is utilized to update the deep motion model parameters ($v_{s_{t}}$, $a_{s_{t}}$) by Equations (9) and (10).

### 3.5. A Novel Model Update Strategy

**Figure 4:**
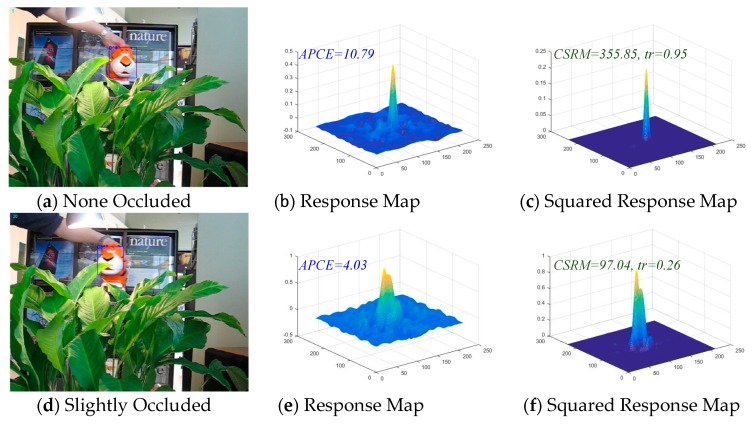

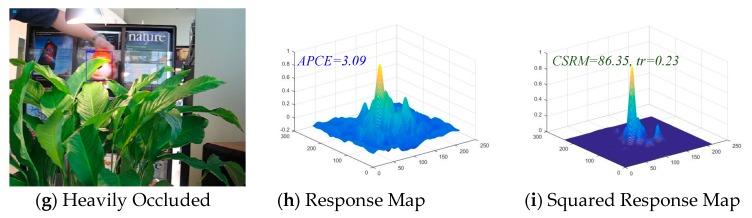
The Confidence of the Squared Response Map (CSRM) in the proposed MACF comparing with the Average Peak-to-Correlation Energy (APCE) of the response map. The example frames are from the sequence “Tiger1” on OTB-100 benchmark. The higher value of the CSMR, the more confident the response map is. The value of parameter *tr* determine the adaptive learning rate which compute by Equation (20). From the figure, the gap of CSMR is larger than APCE between the slightly occluded, heavily occluded and none occluded target.

After the study of Average Peak-to-Correlation Energy (APEC) in [42], a novel confidence function (19) of the responding map is proposed in the MACF algorithm in this paper. In [42], APEC is defined as $APEC=R_{\max}/E\left(R\right)$, here, $R_{\max}$ denotes the max value of the response scores, and E(R) denotes the expected value of the response scores. APCE indicates the fluctuated degree of response maps and the confidence level of the detected targets. Figure 4b,e,h illustrate that if the target apparently appears in the detection scope, there is a sharper peak in the response map and the value of APEC becomes smaller. On the contrary, if the object is occluded, the peak in response map appears smoother, and the relative value of APEC becomes larger.

Unlike the APCE, the proposed method in this article squared the value of response map (the proof is given in Appendix A) and then calculated the value of Confidence of Squared Response Map (CSRM). CSRM stands for the fluctuated degree of the response maps and the confidence level of the detected targets. The numerator of the CSRM represents the peak of the response map, and the denominator of CSRM represents the mean square value of the response map. Figure 4c,f,i illustrate that if the target is not occluded or contaminated, the corresponding response map presents a sharp peak. It is concluded that when the peak value is larger and the mean square value is smaller, and the result is that the corresponding CSRM value is larger. On the contrary, if the target is occluded or contaminated, the corresponding response map will present a smoother peak and even multiple peaks. It could be concluded that when the peak value is smaller and the mean square value is larger, and the result is that the corresponding CSRM value is smaller. This increases the gap between the confidence response and the diffident response as shown in Figure 4, making it easier to find the threshold between them. Consequently, a threshold is set to distinguish whether the target is occluded or contaminated and an adaptive learning rate η is set by Equation (20) to prevent the model from being corrupted. In addition, Equation (20) is effective and accurate for model learning which can be readily and neatly integrated into DCF-based trackers to improve the tracking performance.

(19) $$CSRM_{t}=\frac{{\left|R_{\max}^{2}-R_{\min}^{2}\right|}^{2}}{\frac{1}{MN}\sum_{i=1}^{M}\sum_{j=1}^{N}{\left|R_{ij}^{2}-R_{\min}^{2}\right|}^{2}}\;$$

(20) $$\left\{ \begin{array}{l} tr_{t}=\frac{CSRM_{t}}{CSRM_{0}}\\ \eta_{t}=\eta_{0},\;tr_{t}>tr_{0}\\ \eta_{t}=\eta_{0}\cdot tr_{t},others \end{array}\right.\;$$

where, $CSRM_{0}$ is the Confidence of the Squared Response Map in the initial frame where the response is identified as the most confidence response, $CSRM_{t}$ is the confidence of the squared response map in the *t*-th frame, and $tr_{0}$ is the threshold to decide the learning rate. In Equation (19), the response map R is a two-dimensional M∗N matrix.


**Algorithm 1. MACF tracking algorithm**
**Input:** **1:** Image $I_{t}$. **2:** Predicted target position $PP_{t}$ and scale $Ps_{t}$ in previous frame.
**Output:**
 **1:** Detected target position $P_{t}$ and scale $S_{t}$ in current frame. **2:** Predicted target position $PP_{t+1}$ and scale $Ps_{t+1}$ subsequent frame.**Loop:** **1:** Initialize the Translation model $A_{1,trans}$, $B_{1,trans}$ and Scale model $A_{1,scale}$, $B_{1,scale}$ in the first frame by Equations (3) and (4), and initialize the Confidence of the Squared Response Map $CSRM_{0}$ in the initial frame by Equation (19). **2: for**
$t\in \left[2,t_{f}\right]$
**do**. **3:**  **Position detection and prediction:**
 **4:**    Extract pending sample feature $Z_{t,trans}$ from $I_{t}$ at $PP_{t}$ and $Ps_{t}$. **5:**    Compute correlation scores $Y_{t,trans}$ by Equation (8). **6:**    Set $P_{t}$ to the target position that maximizes $Y_{t,trans}$. **7:**    Predict the position $PP_{t+1}$ of the target of subsequent frame by joint Equations (11) and (17). **8:**  **Scale detection and prediction:** **9:**    Extract pending sample feature $Z_{t,scale}$ from $I_{t}$ at $P_{t}$ and $Ps_{t}$. **10:**    Compute correlation scores $Y_{t,scale}$ by Equation (8). **11:**    Set $S_{t}$ to the target scale that maximizes $Y_{t,scale}$. **12:**    Predict the position $Ps_{t+1}$ of the target of subsequent frame by joint Equations (11) and (17). **13:**  **Model update:** **14:**    Compute the Confidence of the Squared Response Map $CSRM_{t}$ in current frame by Equation (17). **15:**    Compute the adaptive learning rate $\eta_{t}$ by Equation (18). **16:**    Extract sample features $X_{t,trans}$ and $X_{t,scale}$ from $I_{t}$ at $P_{t}$ and $S_{t}$. **17:**    Update motion parameters ($v_{x_{t}}$, $v_{y_{t}}$, $v_{s_{t}}$), ($a_{x_{t}}$, $a_{y_{t}}$, $a_{s_{t}}$) by Equations (9) and (10). **18:**    Update Kalman filters by Equation (18). **19:**    Update the translation model $A_{t,trans}$, $B_{t,trans}$ by adaptive learning rate $\eta_{t}$. **20:**    Update the scale model $A_{t,scale}$, $B_{t,scale}$ by adaptive learning rate $\eta_{t}$. **21:**  **Return**
$P_{t}$, and $PP_{t+1}$, $Ps_{t+1}$. **22: end for**.

## 4. Experiments and Results

In this section, firstly, the implement details and parameter settings are introduced clearly. Then the comprehensive experiments have been tested on the popular benchmark OTB-50, OTB-100 and UAV video, and the results have demonstrated that our MACF approach surpasses most of the state-of-the-art methods.

### 4.1. Implement Details

All the methods compared in this paper are implemented in MATLAB R2016a, and all experiments run on an INTEL i3-3110 CPU with 6 GB memory.

**State-of-the-art trackers:** for other trackers compared to our MACF tracker in this paper, we follow the parameter settings in their papers.

**Trackers proposed in this paper:** Introduced in Section 3.1, the FDSST is employed as the basic tracker. Thus, all parameters of FDSST remain the same as in the paper [16] except for the regularization term λ, learning rate η, search region padding, and scale factor α. In our proposed trackers, the regularization term parameter is set to λ=0.02, the padding region is set to padding=1.8, the scale factor is set to a=1.03 and the adaptive learning rate is calculated from Equation (20) with a threshold $tr_{0}=0.6$. For two-dimensional translation Kalman Filter, the covariances of motion and measured noise in Equations (12) and (13) are set to Q=[25,10,1], R=25. In the one-dimensional scale Kalman Filter, the covariances are set to Q=[2.5,1,0.1], R=2.5. However, there are some different parameter settings about the adaptive learning rate enable parameter, the Kalman position filter enable parameter, the Kalman scale filter enable parameter and the instantaneous motion estimation enable parameter. As described in subsequent Section 4.2, in the proposed MACF tracker, these parameters are respectively set to (1, 1, 1, 1). In the IME_CF tracker, these parameters are respectively set to (0, 0, 0, 1). In the KE_CF tracker, these parameters are respectively set to (0, 1, 1, 0). In the ALR_CF tracker, these parameters are respectively set to (1, 0, 0, 0).

### 4.2. Ablation Experiments

To validate the effectiveness of the strategy proposed in this paper, an ablation experiment is performed on OTB-50, and the MACF is compared with the standard FDSST introduced in Section 2, based on instantaneous motion estimation CFs (IME_CF) discussed in Section 3.1, based on Kalman filters CFs (KF_CF) described in Section 3.2 and based adaptive learning rate CFs (ALR_CF) proposed in Section 3.5. Obviously, Table 1 indicates that the proposed schemes all achieved varying degrees of the tracking performance improvement compared to the standard FDSST. Overall, the proposed MACF achieves a gain of 2.3%, 4.8% and 4.1% in OPE, TRE and SRE, respectively, of LET at 20 pixels and a gain of 1.7%, 1.4% and 2.9% in OPE, TRE and SRE, respectively, of OT at 0.5 compared to the standard FDSST. Furthermore, the proposed MACF run at a real-time speed of 51 FPS in my i3-3110 CPU. However, the strategy of adaptive learning rate achieves the best results instead of our fused MACF. That’s because motion-aware strategy is more suitable to track the target of fast motion in a gradient background. Nevertheless, most video sequences on OTB-50 dataset are with the background of dramatic changes.

### 4.3. Experiment on OTB-50

OTB-50 is an influential benchmark with 50 sequences which are all labeled manually. The proposed MACF is evaluated on this dataset and compared to 11 state-of-the-art trackers from the works: Tracking-Learning-Detection (TLD) [2], DSST [17], FDSST [16], Compressive Tracking (CT) [20], exploiting the Circulant Structure of tracking-by-detection with Kernels (CSK) [21], high-speed tracking with Kernelized Correlation Filters (KCF) [22], Long-term Correlation Tracking (LCT) [45], Locally Orderless Tracking (LOT) [48], Least Soft-threshold Squares tracking (LSS) [49], robust visual tracking via Multi-Task sparse learning (MIT) [50], Distribution Fields for Tracking (DFT) [19]. Only the ranks for the top eight trackers are reported.

**Figure 5:**
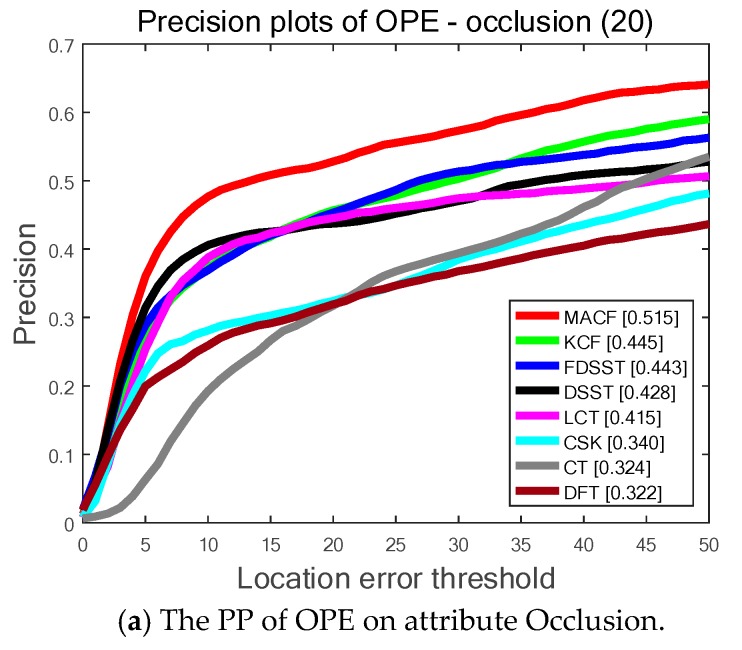

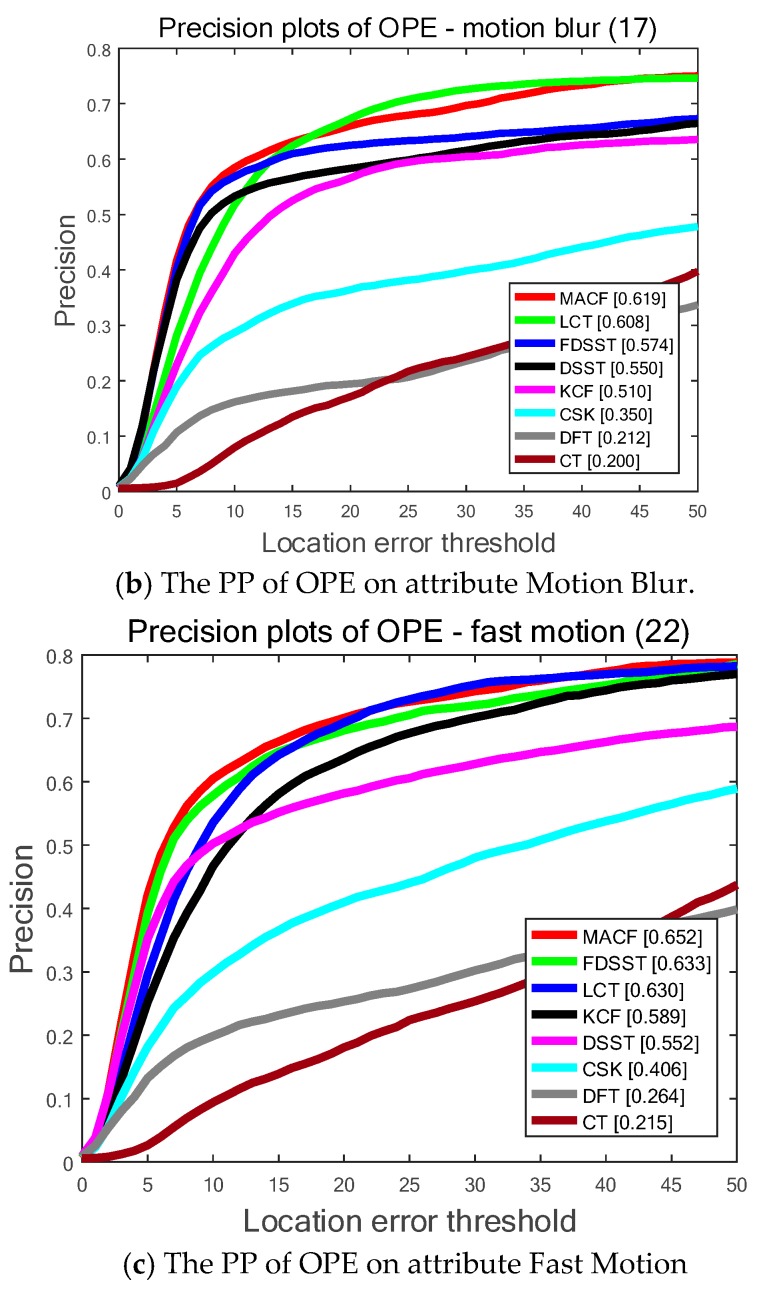
The Precision Plots (PP) of One Pass Evaluation (OPE) on OTB-50 benchmark for the top eight trackers determined by 3 different attributes: occlusion, motion blur and fast motion. Among the top eight trackers our MACF obtains the best results on all 3 attributes.

As is shown in Figure 5, the proposed MACF obtains the top ranks 51.5%, 61.9% and 65.2% among the top eight trackers in 3 different attributes of occlusion, motion blur and fast motion and significantly outperforms the standard FDSST. In other words, the proposed adaptive learning rate scheme is accurate and robust for tracking when the target is occluded or blurred. Furthermore, the proposed motion-aware strategy can effectively track the target of fast motion.

**Figure 6:**
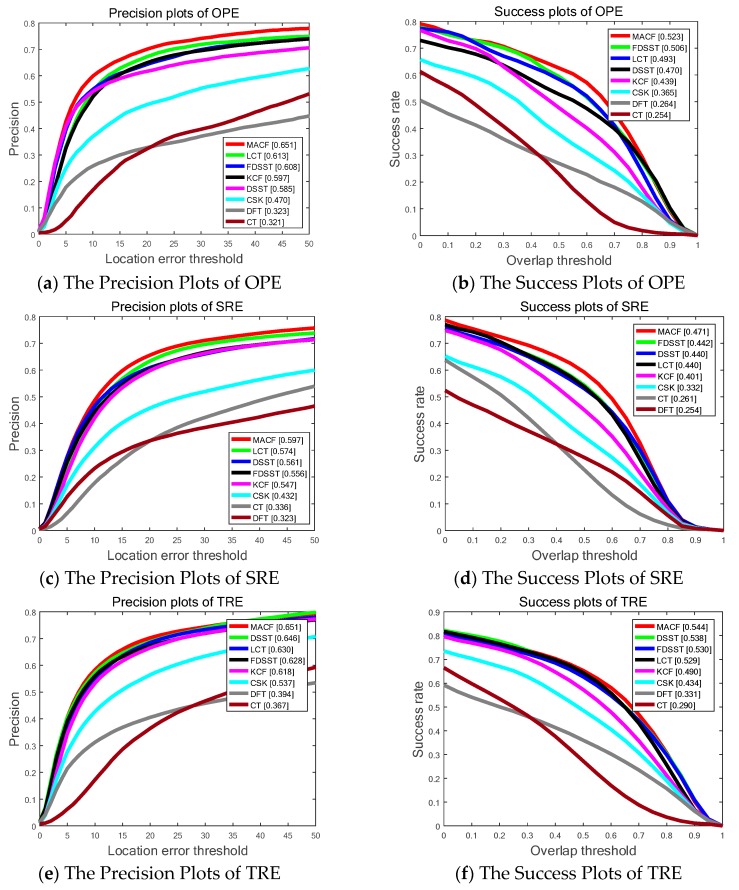
The Success Plots (SP) and Precision Plots (PP) of One Pass Evaluation (OPE), Temporal Robustness Evaluation (TRE) and Spatial Robustness Evaluation (SRE) using by Overlap Threshold (OT) and Location Error Threshold (LET) comparing MACF with the state-of-the-art trackers on OTB-50 benchmark. The ranks for the top 8 trackers are reported with the Area Under the Curve (AUC) marked in brackets.

Figure 6 and Table 2 show the SP of OPE, TRE, and SRE utilizing the LET. The PP of OPE, TRE and SRE using OT with the total 50 sequences on OTB-50 are also shown in Figure 6. Generally, the proposed MACF acquires the best results of the top eight trackers including 65.1%, 59.7% and 65.1% in OPE, TRE and SRE, respectively, of LET at 20 pixels and 52.3%, 47.1% and 54.4% in OPE, SRE and TRE, respectively, of OT at 0.5. Furthermore, the proposed MACF achieves a visibly gain of 4.3%, 4.1% and 2.3% in OPE, SRE and TRE, respectively, of LET at 20 pixels and a gain of 1.7%, 2.9% and 1.4% in OPE, SRE and TRE, respectively, of OT at 0.5 compared to the standard FDSST.

### 4.4. Experiment on OTB-100

OTB-100 is a more challenging benchmark with 100 sequences which are extended by OTB-50. The proposed MACF is evaluated on this dataset and compared to 11 state-of-the-art trackers from the works: TLD [2], DSST [17], FDSST [16], CT [20], CSK [21], KCF [22], LCT [45], LOT [48], LSS [49], MIT [50], DFT [19]. Only the ranks for the top eight trackers are reported.

**Figure 7:**
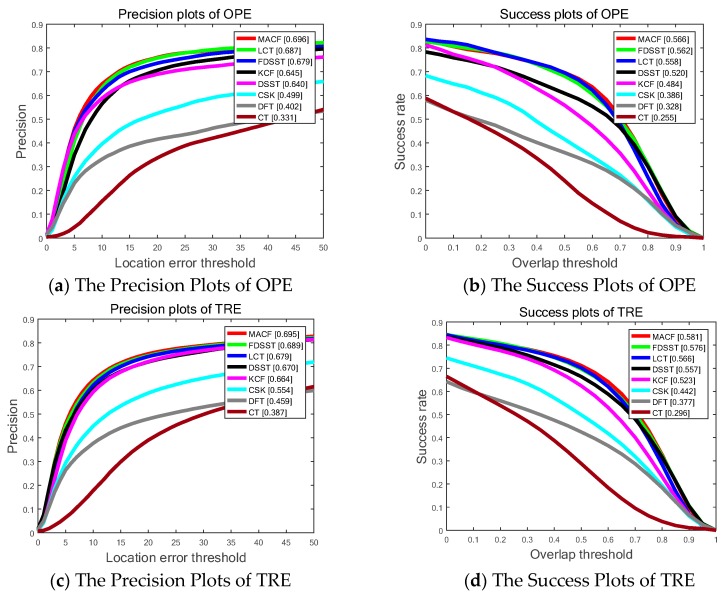

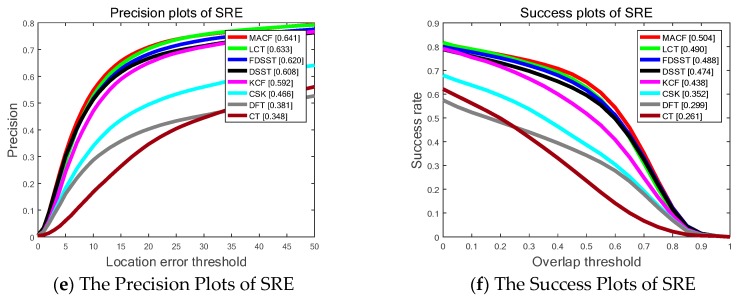
The Success Plots (SP) and Precision Plots (PP) of One Pass Evaluation (OPE), Temporal Robustness Evaluation (TRE) and Spatial Robustness Evaluation (SRE) using by Overlap Threshold (OT) and Location Error Threshold (LET) comparing the MACF with the state-of-the-art trackers on OTB-100 benchmark. In addition, the ranks for the top 8 trackers are reported with the Area Under the Curve (AUC) marked in brackets.

Figure 7 shows SP of OPE, TRE, and SRE utilizing the LET. The PP of OPE, TRE and SRE using OT with the whole 100 sequences on OTB-100 are shown in Figure 5 as well. Overall, the proposed MACF obtain the top ranks of the top eight trackers including 69.6%, 69.5% and 64.1% in OPE, TRE and SRE, respectively, of LET at 20 pixels and 56.6%, 58.1% and 50.4% in OPE, TRE and SRE, respectively, of OT at 0.5. In addition, the proposed MACF achieves a gain of 1.9%, 0.7% and 1.8% in OPE, TRE and SRE, respectively, of LET at 20 pixels and a gain of 0.5%, 0.5% and 1.7% in OPE, TRE and SRE, respectively, of OT at 0.5 compared to the standard FDSST. However, compared to the experiment on OTB-50, the gains go down due to the extent of 50 video sequences are more challenging with dynamic background. Hence, the additional experiments are conducted on the UAV video in Section 4.6 to validate the accurate and robust gains of the MACF on the video streams with static background. 

Table 3 shows the PP of TRE for the top eight trackers determined by 11 different attributes. Among the top eight trackers, the proposed MACF obtains the best results on 8 out of 11 attributes of TRE. Table 4 shows the PP of OPE for the top eight trackers determined by 11 different attributes. Of the top eight trackers the proposed MACF acquires the best ranks on 9 of the 11 attributes of OPE. Table 5 demonstrates the PP of SRE for the top eight trackers determined by 11 different attributes. Of the top eight trackers the proposed MACF achieves the best results on 7 out of 11 attributes of SRE.

**Figure 8:**
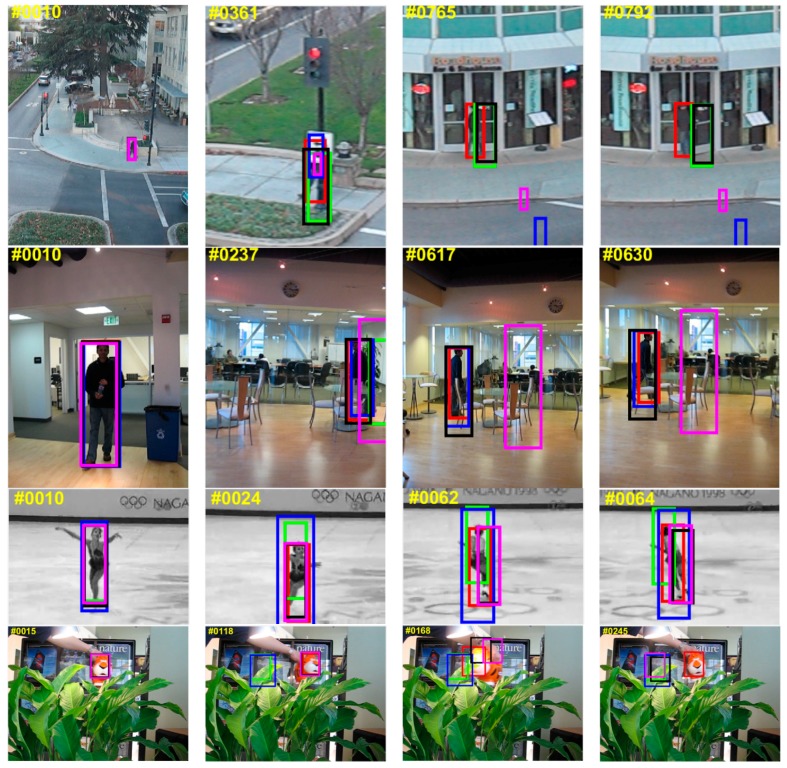
The representative frames from four videos successfully tracked by the MACF (in red) compared to the top 5 trackers including FDSST (in green), LCT (in blue), DSST (in black) and KCF (in purple). From top to bottom, the sequences are “Human6”, “Human2”, “Skater1” and “tiger1” on the OTB-100 benchmark.

Figure 8 qualitatively evaluates the representative frames from four videos successfully tracked by the MACF compared to the top five trackers. From the example frames of Skater1 (the situation of fast motion), it is obvious that the proposed MACF approach performs better than the other four trackers during fast motion and it can be seen from the frames of “Human2” (the situation of occlusion), “Human6” (the situation of occlusion and scale changing greatly), and “Tiger1” (the situation of fast motion and occlusion), the proposed MACF approach is more accurate and robust of the five state-of-the-art trackers when the target is occluded.

### 4.5. Comparation on Raw Benchmark Results

The proposed MACF algorithm is compared to Efficient Convolution Operators for tracking (ECO) [51], Multi-Domain convolutional neural Networks for visual tracking (MDNet) [52], Structure-Aware Network for visual tracking (SANet) [53], Continuous Convolution Operators for visual Tracking (C-COT) [54], Fully-Convolutional Siamese networks for object tracking (SiamFC_3s) [55], Multi-task Correlation Particle Filter for robust object tracking (MCPF) [56], Deep learning features based SRDCF (DeepSRDCF) [26], ECO based on Hand-Crafted features (ECO-HC) [51], Discriminative Correlation Filter Tracker with Channel and Spatial Reliability (CSR-DCF) [25] and FDSST [16] on the raw benchmark results. In addition, all the raw benchmark results are open source on the web. Furthermore, the proposed MACF framework is integrated into the ECO-HC tracker (ECO-HC+MACF) and have been tested on the datasets of OTB-50 and OTB-100. The implementation codes are also open source in our Github https://github.com/YijYang/MACF-ECO_HC.

As shown in Table 6, the fused ECO-HC + MACF tracker achieves a gain of 1.5% and 3.2% in SP and PP of OPE on OTB-50 and a gain of 1.3% and 1.9% in SP and PP of OPE compared to the ECO-HC standard FDSST. In addition, it runs at a real-time speed of 19 FPS compared to the ECO-HC tracker with a speed of 21 FPS. Hence, it indicates that the proposed MACF can be integrated easily and flexibly into other visual tracking algorithms, and with little loss of real-time performance while improving the accuracy. Most trackers based on deep learning features are more accurate than the proposed MACF method. However, these trackers usually have a lower running speed than MACF except SiamFC_3s method which runs at 86 FPS on a GPU. The proposed MACF achieves a trade-off between the tracking speed and the accuracy. Hence, it is suitable for the embedded real-time systems (for instance, UAV surveillance or unmanned vehicles) which have strict memory and speed limitation.

### 4.6. Experiment on UAV Video

#### 4.6.1. Materials and Conditions

The UAV video is taken by a high-definition camera without calibration in the mobile phone. The tested UAV is a high-effective drone from Attop company. The specific parameters of the camera and UAV are illustrated in the Table 7. The UAV video is converted to multi-frame images which have the format of JPG file with three channels, and its resolution is 480 × 640 pixels. In the further research, if the camera for experiment is calibrated, the relative experiment results will be improved [57,58].

#### 4.6.2. Results and Analysis

**Figure 9:**
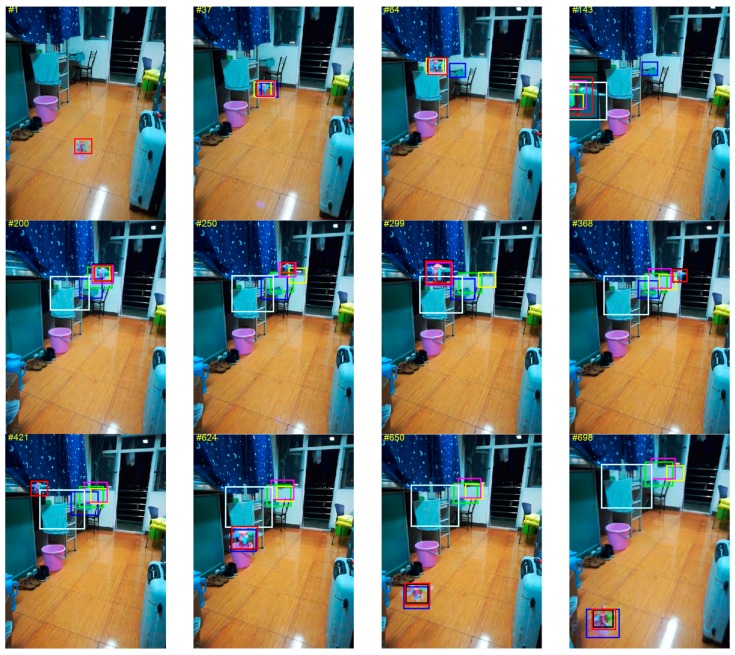
The qualitative experiment comparing the MACF (in red) with state-of-the-art trackers ECO-HC (in blue), BACF (in cyan), STC (in white), Staple (in green), SRDCF (in black), DAT (in yellow) and FDSST (in pink) on UAV video sequence with static background.

As mentioned above, our adaptive learning rate compute by CSRM scheme is greatly suitable for the scenes of occlusion, motion blur, defocus blur and so on when the appearance model of the target is corrupted. Therefore, it can obtain significant gains on OTB-50 and OTB-100. Nevertheless, the motion-aware scheme proposed in this paper is more propitious to the video sequences with static background and target of fast motion. Hence, in order to validate this point, the MACF is compared with the state-of-the-art trackers including Efficient Convolution Operators with HOG feature and Color name feature (ECO-HC) [51], Background-Aware Correlation Filters (BACF) [14], fast tracking via Spatio-Temporal Context learning (STC) [28], Sum of Template And Pixel-wise LEarners (Staple) [27], learning Spatially Regularized Discriminative Correlation Filters (SRDCF) [26], Distractor-Aware Tracking (DAT) [7] and FDSST [16] on the test video which include the target of UAV of fast motion with static background. The results have been shown in Figure 9, which demonstrate that the proposed MACF is more accurate and robust in scale and translation detection when tracking a fast-moving target. It runs at a high speed of 56 FPS.

**Figure 10:**
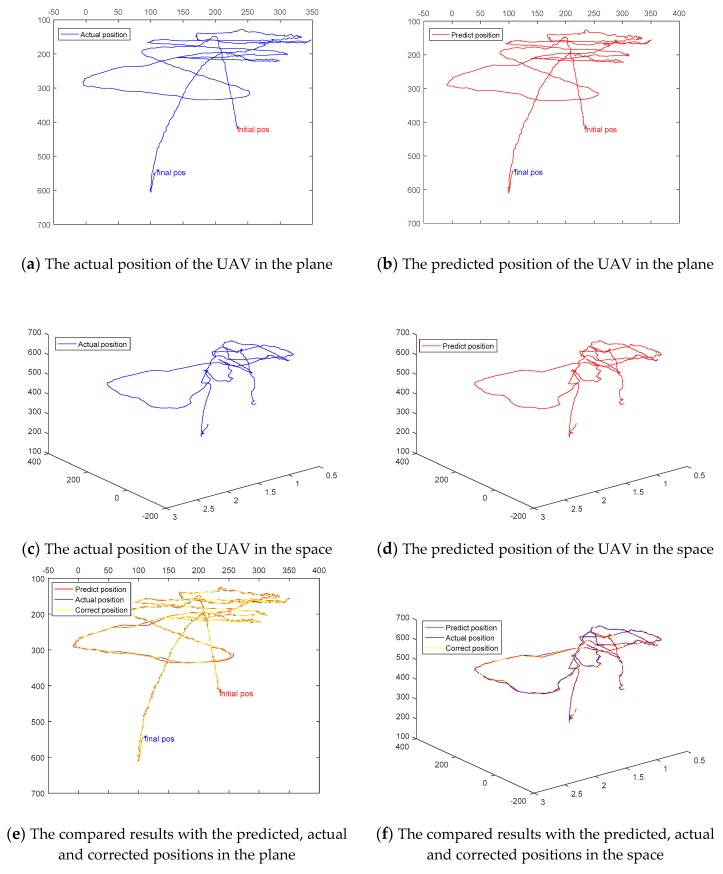
Illustration of the accuracy of the predicted position and scale of the UAV. Here, the Actual position (in blue) is the actual UAV position which is calibrated by manual in the video, the Predict position (in red) is predicted by the instantaneous motion estimation method and the Correct position (in green) is filtered position by Kalman filters. (**a**,**b**) indicate respectively in-plane predicted and actual positions. (**c**,**d**) show apart 3D predicted and actual positions where the scale represents the dept motion. (**e**,**f**) display the overall results.

Figure 9 and Table 8 indicate that the proposed MACF tracker outperforms most of state-of-the-art trackers when undergoes the situation of fast motion. Figure 10 shows the predicted trajectory by the MACF approach is almost coincides with the actual trajectory. It illustrates our motion-aware strategy is accurate for predicting the position and scale of fast-moving target with a static background. As shown in Figure 10a,b, there are still small burrs in the predicted trajectory. However, after correcting by Kalman filters, the trajectory becomes smoother and more accurate as shown in Figure 10e,f.

## 5. Conclusions

In this paper, a novel tracking framework called MACF is proposed in detail, which fuses the motion cues with the FDSST algorithm for accurately estimating the position and scale of the target. The proposed approach utilizes the instantaneous motion estimation method to predict the position and scale of the target in the next frame. The optimal Kalman Filters are employed to filter noises, and then the FDSST tracker is used to detect the position and scale based on the predictions. Moreover, an improved confidence function of response map is further proposed to determine whether the results of detection are accurate enough to update. Then an adaptive learning rate is set according to the confidence function to prevent model corrupted by occlusions. Furthermore, the proposed MACF framework is flexible and can be readily incorporated into other visual tracking algorithms. Numerous experiments on popular benchmark OTB-50, OTB-100 and UAV video indicate that the proposed MACF achieve a significant improvement among the compared trackers. In this work, the situation where the target is occluded is detected by utilizing the confidence function. Then it prevents model drifting by reducing the learning rate. It is suitable for handling the situations of incomplete occlusions. When the target is severely occluded or completely occluded, the proposed MACF sets the learning rate to 0, hence, the model of the target is not be degraded by occlusions. However, if the target comes out of the other side of the occlusion object and moves out of the current search area, the tracking will fail. Therefore, in future work, a re-detect method is expected to track the target when the target is severely occluded or completely occluded to ensure robust tracking. For instance, when the object is completely occluded, the search area should be extended, and the position and scale of the target can be predicted by the previous velocity and acceleration until the target is re-detected judging by the confidence function.

## Figures and Tables

**Table 1:** The comparison of ablation results on OTB-50 dataset. Clearly, the success plots (SP) of one pass evaluation (OPE), temporal robustness evaluation (TRE), and spatial robustness evaluation (SRE) utilizing the location error threshold (LET) and the precision plots (PP) of OPE, TRE and SRE using overlap threshold (OT) and the tracking speed are shown in the table below. And the best results are in red and the second results are in blue.

Trackers	Precision Plots (AUC%)			Success Plots (AUC%)			Speed (FPS)
	OPE	SRE	TRE	OPE	SRE	TRE	
FDSST	62.8	55.6	60.8	50.6	44.2	53.0	49
IME_CF	63.7	58.6	63.3	52.6	46.7	53.7	48
KF_CF	64.5	59.3	65.5	54.4	46.9	54.0	46
ALR_CF	65.0	61.1	66.6	54.6	48.6	54.6	55
MACF	65.1	59.7	65.6	52.3	47.1	54.4	51

**Table 2:** The Success Polts (SP) and Precision Plots (PP) of One Pass Evaluation (OPE) for the proposed MACF and the other 7 top trackers on the OTB-50 dataset. The best results are highlighted in red and the second results are highlighted in blue.

Trackers	OPE		SRE		TRE	
	SP (%)	PP (%)	SP (%)	PP (%)	SP (%)	PP (%)
MACF	52.3	65.1	47.1	59.7	54.4	65.1
FDSST	50.6	60.8	44.2	55.6	53.0	62.8
LCT	49.3	61.3	44.0	57.4	52.9	63.0
DSST	47.0	58.5	44.0	56.1	53.8	64.6
KCF	43.9	59.7	40.1	54.7	49.0	61.8
CSK	36.5	47.0	33.2	43.2	43.4	53.7
CT	25.4	32.1	26.1	33.6	29.0	36.7
DEF	26.4	32.3	25.4	32.3	33.1	39.4

**Table 3:** Success plots of Temporal Robustness Evaluation (TRE) for the MACF and the other 7 top trackers on different attributes: scale variation (SV), illumination variation (IV), out-of-plane rotation (OPR), occlusion (OCC), background cluttered (BC), deformation (DEF), motion blur (MB), fast motion (FM), in-plane rotation (IPR), out-of-view (OV), and low resolution (LR). The last column is the Area Under the Curve (AUC). The best results are in red and the second results are in blue.

Trackers	SV	IV	OPR	OCC	BC	DEF	MB	FM	IPR	OV	LR	AUC
MACF	64.6	68.4	64.9	65.6	70.8	64.3	62.1	61.0	65.8	55.6	72.1	69.5
FDSST	62.6	68.0	63.4	63.5	71.4	61.6	63.7	62.5	65.2	49.4	69.9	67.7
LCT	61.3	67.4	64.1	62.1	68.9	63.5	60.8	57.9	65.3	45.8	66.8	68.6
DSST	62.0	67.5	61.9	60.2	67.4	60.8	56.8	54.3	63.6	46.4	68.3	64.7
KCF	60.5	65.2	63.1	60.4	71.6	60.8	56.3	57.0	63.4	46.5	65.1	63.7
CSK	50.3	54.6	52.0	48.7	57.0	51.4	42.7	41.7	52.9	33.5	54.7	55.7
CT	38.4	35.8	40.9	38.2	38.3	39.9	22.9	25.9	39.9	31.8	49.3	38.7
DEF	38.9	43.5	46.1	43.5	47.6	45.7	35.2	34.9	45.3	29.4	41.6	46.0

**Table 4:** Success plots of One Pass Evaluation (OPE) for the MACF and the other 7 top trackers on different attributes: SV, IV, OPR, OCC, BC, DEF, MB, FM, IPR, OV, and LR. The last column is the AUC. The best results are in red and the second results are in blue.

Trackers	SV	IV	OPR	OCC	BC	DEF	MB	FM	IPR	OV	LR	AUC
MACF	66.2	71.8	65.5	63.5	72.6	62.5	63.6	63.9	67.0	57.3	65.2	69.6
FDSST	61.8	68.4	62.8	59.5	70.5	58.5	61.6	63.2	66.9	50.4	64.7	68.8
LCT	61.9	67.8	66.6	60.2	66.1	61.6	60.2	62.0	69.9	52.0	64.3	68.1
DSST	59.3	67.5	60.6	56.6	63.8	52.8	52.0	51.0	62.8	43.1	63.6	66.9
KCF	58.2	64.2	62.9	60.0	65.2	58.6	55.3	58.1	63.8	48.0	62.3	66.5
CSK	44.2	47.3	46.7	42.0	52.7	42.5	34.9	38.7	49.5	27.7	43.8	49.3
CT	32.8	29.7	35.6	32.4	35.8	31.5	20.7	21.1	34.9	30.8	40.3	33.0
DEF	34.5	39.7	43.0	41.6	43.1	40.4	27.6	30.5	41.4	34.4	41.9	40.6

**Table 5:** Success plots of Spatial Robustness Evaluation (SRE) for the MACF and the other 7 top trackers on different attributes: SV, IV, OPR, OCC, BC, DEF, MB, FM, IPR, OV, and LR. The last column is the AUC. The best results are in red and the second results are in blue.

Trackers	SV	IV	OPR	OCC	BC	DEF	MB	FM	IPR	OV	LR	AUC
MACF	60.6	64.5	59.8	58.7	63.4	56.1	56.9	57.9	62.3	51.7	67.5	64.1
FDSST	56.9	60.0	57.8	55.7	62.5	51.2	56.1	58.4	61.4	44.2	64.1	61.8
LCT	57.4	61.8	62.0	56.5	59.7	58.8	52.7	49.9	64.8	44.7	62.7	63.3
DSST	56.8	62.7	56.8	53.7	60.9	50.1	49.0	55.1	59.6	42.5	64.3	60.6
KCF	53.7	58.9	57.0	52.9	60.1	53.8	48.8	53.1	58.3	39.7	56.9	59.4
CSK	41.2	44.7	45.0	41.5	45.7	38.9	33.4	36.4	46.8	28.9	45.9	46.2
CT	35.0	30.9	35.8	33.7	31.6	32.8	22.2	24.6	36.4	30.2	42.6	34.4
DEF	31.9	34.8	38.7	36.0	40.3	35.3	28.9	30.4	40.3	28.3	34.5	37.8

**Table 6:** SP and PP of OPE for the proposed MACF, ECO-HC + MACF and the other 10 top trackers on the raw benchmark results of OTB-50 and OTB-100. The last column is the performance of Real-Time and the results are from the original paper, not tested on the same platform. The column of Deep Learning indicates whether the tracker is based on deep learning features. The best results are in red and the second results are in blue.

Trackers	OTB-50		OTB-100		Deep Learning	Real Time (FPS)
	SP of OPE (%)	PP of OPE (%)	SP of OPE (%)	PP of OPE (%)		
ECO	64.3	87.4	69.4	91.0	Y	N (6)
MDNet	64.5	89.0	67.8	90.9	Y	N (1)
SANet	--	--	69.2	92.8	Y	N (1)
C-COT	61.4	84.3	67.1	89.8	Y	N (0.3)
SiamFC_3s	51.6	69.2	58.2	77.1	Y	Y (86)
MCPF	58.3	84.3	62.8	87.3	Y	N (0.5)
DeepSRDCF	56.0	77.2	63.5	85.1	Y	N (<1)
CSR-DCF	59.7	66.7	59.8	73.3	N	Y (13)
ECO-HC + MACF	60.7	84.6	65.6	87.5	N	Y (19)
ECO-HC	59.2	81.4	64.3	85.6	N	Y (21)
MACF	52.3	65.1	56.6	69.6	N	Y (51)
FDSST	50.6	60.8	56.2	67.9	N	Y (49)

**Table 7:** The parameters of the tested camera and UAV.

Camera Parameters		UAV Parameters	
Aperture size	F2.2	Product number	W5
Number of Pixel	1200 W	Expand Size	15.5 × 15.5 × 10 cm
Size of Pixel	1.25 μm	Color	Red
Focusing speed	0.23 s	Type of Control Signal	Wireless Fidelity (Wi-Fi)
Image dimensions	3	Others	No Antivibration used and No gimbal [59] used

**Table 8:** The Success Plots (SP) and Precision Plots (PP) of One Pass Evaluation (OPE) for the proposed MACF and the other 7 top trackers on the UAV video. The best results are in red and the second results are in blue.

Trackers	MACF	ECO_HC	BACF	STC	STAPLE	SRDCF	DAT	FDSST
**SP of OPE (%)**	100.0	94.8	20.6	31.4	21.8	98.7	23.1	46.8
**PP of OPE (%)**	100.0	98.6	24.1	32.2	29.1	99.5	32.5	52.6

## Appendix A

In this section, the expressions bellow are used to prove that the squared response map has a significant effect on confidence calculation. As described in Section 3.5, the Confidence of the Squared Response Map function (CSRM) is defined as follows:

$$CSRM=\frac{{\left|R_{\max}^{2}-R_{\min}^{2}\right|}^{2}}{\frac{1}{MN}\sum_{i=1}^{M}{\sum_{j=1}^{N}\left|R_{ij}^{2}-R_{\min}^{2}\right|}^{2}}\;$$

The Confidence of Response Map function (CRM) is defined as follows:

$$CRM=\frac{{\left|R_{\max}^{}-R_{\min}^{}\right|}^{2}}{\frac{1}{MN}\sum_{i=1}^{M}{\sum_{j=1}^{N}\left|R_{ij}^{}-R_{\min}^{}\right|}^{2}}\;$$

Hence, the difference between the CSRM and CRM compute by follows:

$$\begin{array}{l} \mathrm{CSRM-CRM}=\frac{{\left|R_{\max}^{2}-R_{\min}^{2}\right|}^{2}}{\frac{1}{MN}\sum_{i=1}^{M}{\sum_{j=1}^{N}\left|R_{ij}^{2}-R_{\min}^{2}\right|}^{2}}-\frac{{\left|R_{\max}^{}-R_{\min}^{}\right|}^{2}}{\frac{1}{MN}\sum_{i=1}^{M}{\sum_{j=1}^{N}\left|R_{ij}^{}-R_{\min}^{}\right|}^{2}}\\ \geq \frac{{\left|R_{\max}^{2}-R_{\min}^{2}\right|}^{}}{\frac{1}{MN}\sum_{i=1}^{M}{\sum_{j=1}^{N}\left|R_{ij}^{2}-R_{\min}^{2}\right|}^{}}-\frac{{\left|R_{\max}^{}-R_{\min}^{}\right|}^{}}{\frac{1}{MN}\sum_{i=1}^{M}{\sum_{j=1}^{N}\left|R_{ij}^{}-R_{\min}^{}\right|}^{}}\\ =\frac{{\left|R_{\max}^{}-R_{\min}^{}\right|}^{}*\sum_{i=1}^{M}{\sum_{j=1}^{N}\left|\left(R_{ij}^{}-R_{\min}^{})*(R_{\max}^{}-R_{\min}^{}\right)\right|}^{}-{\left|R_{\max}^{}-R_{\min}^{}\right|}^{}*\sum_{i=1}^{M}{\sum_{j=1}^{N}\left|R_{ij}^{2}-R_{\min}^{2}\right|}^{}}{\frac{1}{M^{2}N^{2}}\sum_{i=1}^{M}{\sum_{j=1}^{N}\left|R_{ij}^{2}-R_{\min}^{2}\right|}^{}*\sum_{i=1}^{M}{\sum_{j=1}^{N}\left|R_{ij}^{}-R_{\min}^{}\right|}^{}}\\ \geq \frac{{\left|R_{\max}^{}-R_{\min}^{}\right|}^{}*\sum_{i=1}^{M}\sum_{j=1}^{N}\left|\left(R_{ij}^{}-R_{\min}^{})*(R_{\max}^{}-R_{ij}^{}\right)\right|}{\frac{1}{M^{2}N^{2}}\sum_{i=1}^{M}{\sum_{j=1}^{N}\left|R_{ij}^{2}-R_{\min}^{2}\right|}^{}*\sum_{i=1}^{M}{\sum_{j=1}^{N}\left|R_{ij}^{}-R_{\min}^{}\right|}^{}}\\ \geq 0 \end{array}$$

Therefore, CSRM≥CRM and the difference between them increases as the value of $R_{\max}$ increases. Furthermore, the larger value of $R_{\max}$ means the higher confidence score. Hence, this increases the gap between the confidence response and the diffident response.
